# Determination of polysaccharides composition in *Polygonatum sibiricum* and *Polygonatum odoratum* by HPLC-FLD with pre-column derivatization

**DOI:** 10.1016/j.heliyon.2022.e09363

**Published:** 2022-04-30

**Authors:** Sheng Xu, Jianli Bi, Wenfang Jin, Baolei Fan, Chunqi Qian

**Affiliations:** a Hubei University of Science and Technology, China; b Michigan State University, United States

**Keywords:** *Polygonatum sibiricum*, *Polygonatum odoratum*, Monosaccharides, High performance liquid chromatography, Fluorescence detection, Derivatization of p-aminobenzoic acid

## Abstract

A high-performance liquid chromatography-fluorescence detection (HPLC-FLD) method was established for the determination of seven monosaccharides in *Polygonatum sibiricum* and *Polygonatum odoratum*. The polysaccharides were de-esterified, extracted, hydrolyzed and derivatized with p-aminobenzoic acid (PABA) to obtain fluorescently labeled monosaccharide compounds, which were finally detected by HPLC-FLD. Inertsil ODS-3, C18 chromatographic column (250 mm × 4.6 mm, 5 μm) was used for chromatography. The excitation wavelength (E_x_) was 313 nm, and the emission wavelength (E_m_) was 358 nm. Ethyl acetate extraction reduced the peaks of chromatogram and improved the detection sensitivity than other agents. The established method had high sensitivity, strong specificity, good linear relationship and recovery efficiency. The results showed that the roots and fibrous roots of *Polygonatum sibiricum* and *Polygonatum odoratum* contained these seven monosaccharides, and the highest monosaccharide content was mannose. The method of PABA-HPLC-FLD for determination of monosaccharide content in *Polygonatum sibiricum* and *Polygonatum odoratum* was sensitive and accurate. The method established in this work provides a feasible analytical tool for the study of polysaccharides, and the findings on polysaccharides from *Polygonatum sibiricum* and *Polygonatum odoratum* can provide guidance for the natural medicine industry.

## 1 Introduction

*Polygonatum sibiricum Red.* and *Polygonatum odoratum (MilL) Druce* are the dried roots of *Polygonatum sibiricum* and *Polygonatum odoratum* of Liliaceae. They belong to *Polygonatum*, and they are similar in shape and difficult to distinguish. The rhizomes of *Polygonatum sibiricum* and *Polygonatum odoratum* in the same year are shown in Figure S1 in the Supplementary Material. *Polygonatum odoratum* has the efficacy of promoting salivation, moistening lung, nourishing stomach and tonifying kidney, while efficacy of *Polygonatum odoratum* mainly includes building up vital energy, invigorating spleen, moistening lung and tonifying kidney, etc [1]. Both *Polygonatum sibiricum* and *Polygonatum odoratum* are rich in polysaccharides [2, 3, 4], flavonoids [5] and saponins [6, 7], among which polysaccharides are the most important chemical components in *Polygonatum sibiricum* and *Polygonatum odoratum*. *Polygonatum sibiricum* polysaccharide and *Polygonatum odoratum* polysaccharide have high medicinal value. They have good medicinal effects including antibacterial, anti-inflammation [6, 8], antioxidation [7, 9], hypoglycemia [6, 10], antitumor [11, 12] and immunomodulation [6, 13]. The effective components of the two kinds of traditional Chinese medicines are polysaccharides, which have different medicinal values due to their different composition and structure. The analysis of monosaccharide composition in polysaccharides is important to study the structural property and quality control of polysaccharides. Because the monosaccharide composition of the two Chinese medicines is very similar, it is easy to confuse them in process production and clinical use. In order to distinguish the difference in chemical components between the two medicines, a sensitive and reliable polysaccharide detection method needs to be established, which is of great significance to identify the differences in polysaccharide composition and the content of the two medicines.

The main detection methods of polysaccharides are ultraviolet spectroscopy (UV) after phenol sulfuric acid addition [14], high performance liquid chromatography (HPLC-UV) [14, 15, 16], gas chromatography (GC) [17], capillary electrophoresis (CE) [18, 19] and high-performance liquid chromatography-tandem mass spectrometry (HPLC-MS) [20, 21]. Ultraviolet spectroscopy is only suitable for detection of total polysaccharides, making it impossible to distinguish monosaccharide composition of traditional Chinese medicine with complex chemical composition. The sensitivity of gas chromatography is low, making it difficult to detect monosaccharide components with low content. The pretreatment process of capillary electrophoresis is complex with poor repeatability. Mass spectrometry is not suitable for popularization because of its complicated pretreatment and expensive instrument. High performance liquid chromatography (HPLC) is widely used for the detection of effective components in Chinese medicinal materials because of its high sensitivity and good reproducibility. In particular, when fluorescence detector is used, the detection sensitivity is similar to that of mass spectrometry, and the instrument cost is low, making it suitable for detection of compounds containing fluorescent groups [22].

**Figure 1:**
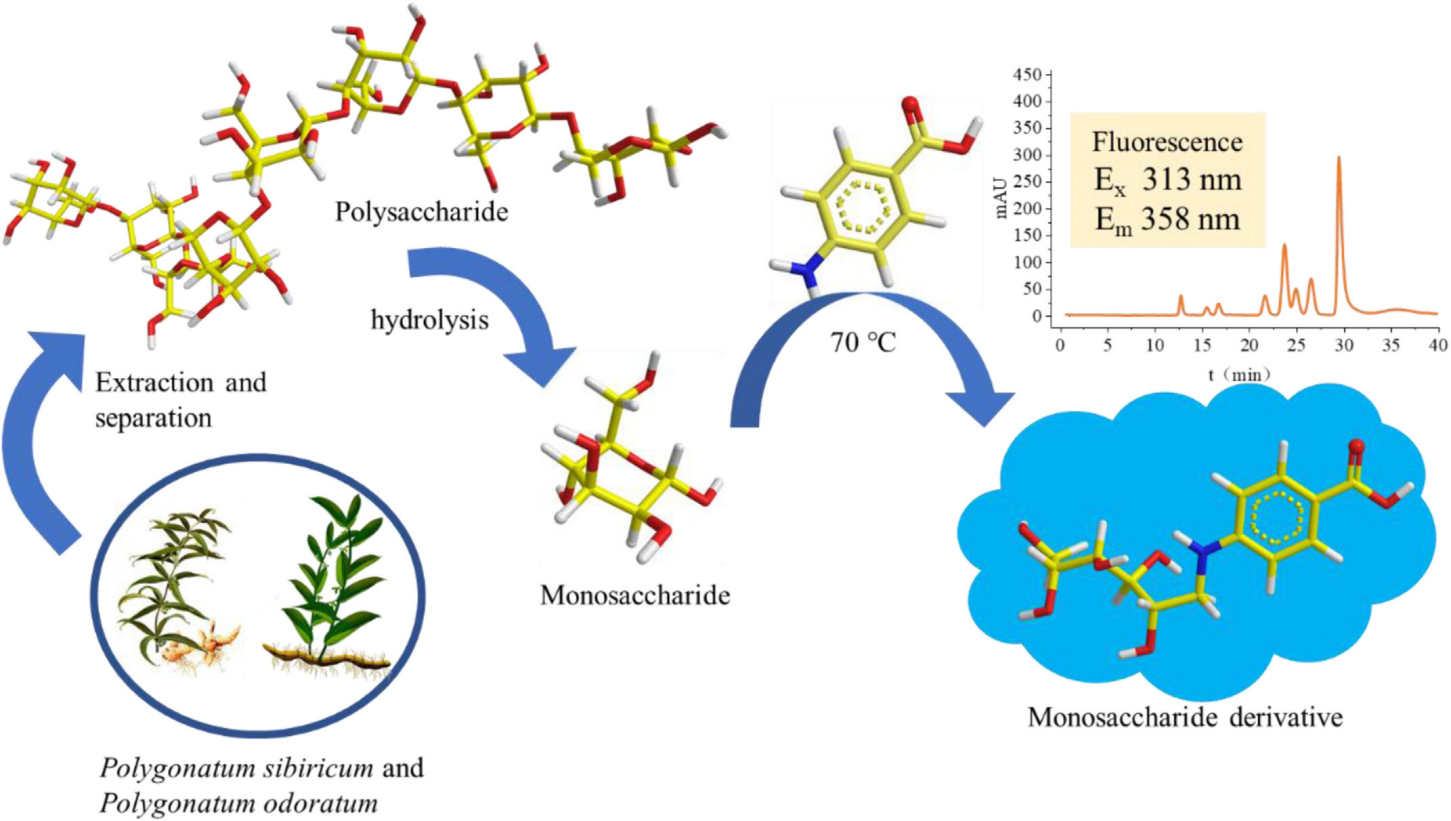
Overview of fluorescence detection of *Polygonatum sibiricum* and *Polygonatum odoratum* monosaccharide derivatives.

Because of the lack of conjugated structural components, polysaccharide compounds have no fluorescence absorption, and it is generally necessary to introduce derivatizing reagents to improve detection sensitivity. At present, the main methods to derivatize monosaccharides utilize 1-phenyl-3-methyl-5-pyrazolone (PMP) [23, 24], p-aminobenzoic acid (PABA) [25] or O-aminobenzoic acid [26, 27]. PMP derivatization is the most widely used derivatizing reagent for monosaccharide detection, which can be used for UV and MS detection of monosaccharide, but the technical challenges such as long analysis time and insufficient detection sensitivity are hard to overcome. O-aminobenzoic acid is hard to produce, so its applicability will be limited when it is used in monosaccharide detection. P-aminobenzoic acid is cheap and easy to obtain with good derivatization efficiency. Its monosaccharide derivative has fluorescence absorption property and good emission efficiency. Using fluorescence to detect chemical components in Chinese medicinal materials can increase detection sensitivity and reduce the interference effect of sample impurities. These features are especially advantageous for compounds with complex chemical components and low content in Chinese medicinal materials. In this study, the native *Polygonatum sibiricum* and *Polygonatum odoratum* will be extracted after ester removal, and the obtained polysaccharides will be hydrolyzed under acidic conditions. The monosaccharides after hydrolyzed will be derivatized by PABA, and the content of monosaccharides in *Polygonatum sibiricum* will be determined by HPLC-FLD. The experimental flow chart is shown in Figure 1. The experimental results will provide basis for determination of chemical components in Chinese medicinal materials by using PABA-derivatized monosaccharides, providing a reference basis for further development and utilization of *Polygonatum sibiricum* and *Polygonatum odoratum*.

## 2 Materials and methods

### 2.1 Materials and instruments

*Polygonatum sibiricum* and *Polygonatum odoratum*, excavated in April, were both produced in Tongcheng County, Xianning City. It was identified as the dried rhizome of *Polygonatum sibiricum* and *Polygonatum odoratum* by Associate Professor Xiao Ruolei from School of Pharmacy, Hubei University of Science and Technology. The reference substances Fructose (Fru), Rhamnose (Rha), Glucose (Glc), Mannose (Man), Galactose (Gal), Xylose (Xyl) and Galacturonic acid (GalUA) were purchased from Aladdin with 98% purity. P-aminobenzoic acid (PABA), sodium cyanoborohydride and tetrabutylammonium hydrogen sulfate (TBAHSO_4_) were all purchased from Rhawn Reagent. Acetonitrile, methanol, chromatographic grade, Wahaha purified water as mobile phase, and other reagents were analytically pure.

Shimadzu LC-16 liquid chromatograph was equipped with RF-10AXL fluorescence detector, Shimadzu Corporation, Japan; UV-2700 ultraviolet spectrophotometer, Shimadzu Corporation, Japan; FA2004B electronic balance, Shanghai yueping scientific instrument co., ltd.; KW-1000DC digital display constant temperature water bath pot, Jiangsu Jintan Yitong Electronics Co., Ltd.; RE-5299 rotary evaporator, Zhengzhou Yarong Instrument Co., Ltd.; SHZ-D (Ⅲ) circulating water type multipurpose vacuum pump, Zhengzhou Boko Instrument Equipment Co., Ltd.; LGJ-10A freeze dryer, Shanghai hefan instrument co., ltd.

### 2.2 Methods

#### 2.2.1 Liquid chromatography conditions

Inertsil ODS-3, C18 column (250 mm × 4.6 mm, 5 μm); The mobile phase was 0.3% formic acid aqueous solution (containing 5 mmol/L ammonium formate and 20 mmol/L TBAHSO_4_) (A)- acetonitrile (B), and 3% isocratic elution. Column temperature 25 °C; The flow rate was 0.5 mL/min; The excitation wavelength (E_x_) was 313 nm, and the emission wavelength (E_m_) was 358 nm. Sample injection volume was 10 μL.

#### 2.2.2 Preparation of derivative reagents

We weighed 0.35 g of PABA and 0.05 g of sodium cyanoborohydride, added 0.5 mL of glacial acetic acid, diluted it to 5 mL with methanol, vortexed them for 1 min and mixed them evenly to obtain PABA derivative reagent of monosaccharide. The mixture was prepared immediately before use.

#### 2.2.3 Preparation of reference substance solution

We accurately weighed a certain amount of reference substance of Fru, Rha, Gal, Glc, Man, Gal, Xyl and GalUA, and dissolved each compound in pure water to prepare a single reference substance stock solution of 0.50 mg/mL. We then prepared a mixed reference substance solution with concentration of 0.50 mg/mL, and stored it for later use.

#### 2.2.4 Extraction of polysaccharides from *Polygonatum sibiricum* and *Polygonatum odoratum*

The samples of *Polygonatum sibiricum* and *Polygonatum odoratum* were washed and drained, cut into slices. Their fibrous roots were chopped, dried under reduced pressure at 50 °C in a vacuum drying oven for 3 d, crushed, and sieved with a 40-mesh sieve to obtain sample powders of *Polygonatum sibiricum* and *Polygonatum odoratum*. We accurately weighed 2 g of each sample powder, added 50 mL of absolute ethanol, reflux extracted at 80 °C for 1 h, performed suction filtration, repeatedly refluxed the filter residue once, took the filter residue by suction filtration, transferred it into a round bottom flask, added 150 mL of water, and performed reflux extraction at 90 °C for 2 h. The filtrate was obtained by suction filtration, and the filtrate was concentrated to 25 mL in a rotary evaporator, and 4-fold the volume of absolute ethanol was added to obtain crude polysaccharide after complete precipitation. The crude polysaccharide was dissolved in distilled water, added by 10 mL of Sevage reagent (chloroform: n-butanol volume ratio 4:1), vortexed and mixed well, centrifuged at 3500 rpm for 5 min. The supernatant was separated, added by 4-fold volume of absolute ethanol, precipitated, washed with absolute ethanol and acetone respectively. The mixture was filtered and dried to obtain *Polygonatum sibiricum* and *Polygonatum odoratum* polysaccharides.

#### 2.2.5 Hydrolysis of polysaccharide

We accurately weighed 10 mg of *Polygonatum sibiricum* and *Polygonatum odoratum* polysaccharide, added 5 mL of 2 mol/L hydrochloric acid aqueous solution, sealed and mixed well, hydrolyzed at 90 °C for 3 h, cooled to room temperature, centrifuged at 3500 rpm for 10 min. The supernatant was taken to obtain *Polygonatum sibiricum* polysaccharide hydrolysate.

#### 2.2.6 PABA derivatization of monosaccharides

We accurately measured 200 μL of polysaccharide hydrolysate, added 200 μL of 0.3 mol/L sodium hydroxide aqueous solution and 200 μL of PABA derivative reagent, derivatized them at 70 °C in water bath for 40 min. After cooling, 1 mL of ethyl acetate was added to extract excessive PABA. The upper layer liquid was extracted for 3 times, the lower layer liquid was diluted to 2 mL with water and passed through 0.45-μm microporous membrane for detection.

In the derivatization process of monosaccharide reference substance, each monosaccharide reference substance was prepared into a mixed reference substance solution of 10 μg/mL, which was treated by the same method as the above-mentioned monosaccharide PABA derivatization method to obtain a reference substance derivative solution of monosaccharide, and analyzed according to the above liquid chromatography conditions. The pretreatment methods of *Polygonatum sibiricum* polysaccharide and *Polygonatum odoratum* polysaccharide were shown in Figure S2 in the Supplementary Material.

#### 2.2.7 Methodology

##### 2.2.7.1 Linear relationship, limit of quantitative and limit of detection

The mixed reference solution was diluted to a certain concentration with methanol, and then diluted to mixed reference solutions containing monosaccharide components of 0.5, 1.0, 5.0, 10.0, 20.0, 50.0 and 100 μg/mL respectively, which were derivatized according to the above derivatization method and analyzed by HPLC system. The linear equations and correlation coefficients of seven monosaccharides were calculated with peak area as ordinate (Y) and reference substance concentration as abscissa (X). The reference standard solution was gradually diluted to a signal-to-noise ratio (S/N) of 10 and a signal-to-noise ratio of 3, respectively, to calculate the limit of quantitation (LOQ) and limit of detection (LOD) of the method.

##### 2.2.7.2 Precision

We took the mixed reference solution with a certain concentration, repeated sample injection for 6 times according to the above chromatographic conditions and calculated the RSD of peak area of each component respectively.

##### 2.2.7.3 Reproducibility

According to the above preparation method of test solution, 6 test solutions were prepared from the same *Polygonatum sibiricum* sample in parallel, and analyzed according to the above chromatographic conditions, and the RSD of peak area of each component was determined.

##### 2.2.7.4 Stability

We took a sample solution from the repeatability test, injected it for analysis at 0, 1, 2, 4, 6, 8, 12 h according to the above chromatographic conditions, and calculated the RSD of peak area of each component to verify the stability.

##### 2.2.7.5 Recovery

We took 2 g (6 parts) of *Polygonatum sibiricum* powder sample with known content, weighed it accurately, put it in a round-bottom flask, added a certain amount of each reference solution accurately. We prepared the test solution with sample addition recovery rate according to the method in item 1.2.3, injected samples for analysis according to the above conditions, and calculated the average recovery of each component.

#### 2.2.8 Data processing

The experimental data was recorded and plotted using origin 2017, the orthogonal experimental table was designed and analyzed using SPSS Statistics 25, and principal component analysis used Simca 13.0 to distinguish *Polygonatum sibiricum* from *Polygonatum odoratum* and related fibrous roots samples.

## 3 Results and analysis

### 3.1 Optimization of derivatizing conditions

#### 3.1.1 Influence of derivatization conditions on derivatives

Comparing the peak conditions of the samples when the amount of derivatization reagent was 50 μL, 100 μL, 200 μL, 300 μL and 400 μL respectively, it was found that the derivatization effect is good when the amount of derivatization reagent was 200 μL. Increasing the amount of derivatization reagent would reduce the peak area of derivatization products. Comparing the peak areas of derivative products under the conditions of derivatization for 10 min, 20 min, 30 min, 40 min, 50 min and 60 min, it was found that the derivatization effect was good at 40 min and increasing the derivatization time might lead to the decomposition of derivative products. The derivatization effects at 40 °C, 50 °C, 60 °C, 70 °C, 80 °C and 90 °C were compared respectively. The results showed that the derivatization effect was good at 70 °C, and the derivative products had high response value, which was suitable for the reaction of derivative products. The experimental results were shown in Figure S3 in the Supplementary Material. The best derivatization condition of monosaccharide was to add 200 μL of PABA derivatization reagent into 200 μL of monosaccharide aqueous solution (10 μg/mL) and derivatize at 70 °C for 40 min.

#### 3.1.2 Optimization of derivatization conditions by orthogonal experiment

By designing orthogonal experiment to optimize the derivatization effect under different conditions under 3 factors and 3 levels, the best derivatization conditions of PABA were obtained. Orthogonal experiment optimization results were designed at different levels of derivatization temperature 60 °C, 70 °C, 80 °C, for derivatization time of 30 min, 40 min, 50 min, and derivatization reagent dosage of 100 μL, 200 μL, 300 μL. The optimized results of orthogonal experiment were shown in Table 1. Under different derivatization conditions, the total areas of chromatographic peaks of monosaccharide derivatives were different. Based on the total peak area, the derivatization effects under different derivatization conditions were compared. The results showed that the optimal temperature, time, and dosage of derivatizing agent were 70 °C, 40 min and 200 μL, respectively. At this time, the total peak areas of the derivatives of the seven monosaccharides were the largest. It can be seen from the table that among the factors affecting the derivatization efficiency, the dosage of derivative had the greatest effect on the derivative result, followed by the reaction time, and the factor that had the smallest effect is the reaction temperature.

**Table 1:** L_9_(3^3^)Orthogonal test results of monosaccharide derivatization in *Polygonatum sibiricum* and *Polygonatum odoratum*.

Number	Time	Temperature	Derivative dosage	Total peak area
1	2	2	2	2.413×10^8^
2	1	1	1	2.285×10^8^
3	3	2	1	2.288×10^8^
4	3	1	2	2.356×10^8^
5	1	3	2	2.326×10^8^
6	2	1	3	2.409×10^8^
7	2	3	1	2.268×10^8^
8	1	2	3	2.351×10^8^
9	3	3	3	2.408×10^8^
K_1_	2.321×10^8^	2.350×10^8^	2.280×10^8^	
K_2_	2.363×10^8^	2.351×10^8^	2.365×10^8^	
K_3_	2.351×10^8^	2.334×10^8^	2.389×10^8^	
R	4.2×10^6^	1.7×10^6^	1.09×10^7^	

#### 3.1.3 Verification experiment

Under the conditions of derivatization time of 40 min, derivatization temperature of 70 °C and dosage of derivatization agent of 200 μL, the average peak area was 2.412×10^8^, with RSD of 3.4%, which indicated that the derivatization effect was good and was suitable for PABA derivatization of seven monosaccharides in *Polygonatum sibiricum* and *Polygonatum odoratum*.

### 3.2 Optimization of HPLC conditions

#### 3.2.1 Determination of detection wavelength

In this experiment, the UV absorption spectrum of monosaccharide derivatives was scanned by UV-vis spectrometer. The maximum absorption wavelength of monosaccharide derivatives was 315 nm as the excitation wavelength (E_x_) of fluorescence spectrum, and the emission wavelengths (E_m_) of seven monosaccharide derivatives were scanned. The fluorescence detection conditions of monosaccharide derivatives were determined as excitation wavelength 313 nm and emission wavelength 358 nm.

#### 3.2.2 Optimization of chromatography separation

After monosaccharide derivatization, acetonitrile -0.05 mol/L phosphate buffer solution (PBS) was used as the mobile phase for determination, which showed multiple chromatographic peaks in liquid chromatography, making it impossible to determine the single chromatographic peak of each monosaccharide. Only one peak appeared in the chromatogram after extraction with ethyl acetate, and this peak appeared before the derivatization reagent. The monosaccharide standards were analyzed by liquid chromatography respectively, and the results showed that the peak positions of the seven monosaccharides were the same.

In addition, after addition of TBAHSO_4_, the peak positions of seven monosaccharides changed from before to after the derivatization agent, and the use of this reagent could increase the separation of monosaccharide derivatives and derivatization reagents. It could also improve the separation of seven monosaccharides. In the experiment, we found that TBAHSO_4_ was an important factor to separate monosaccharides and that the extraction efficiency was another important factor to judge the chromatographic peaks of monosaccharide derivatives. The peak position of monosaccharide derivatives was related to the amount of TBAHSO_4_ and the proportion of water phase. In reference [25], the peak position of fructose was before PABA, which may be caused by the small amount of TBAHSO_4_ or the high concentration of organic phase. Increasing the proportion of TBAHSO_4_ could make the peak position of monosaccharide chromatographic peak behind the derivatization reagent. When the acidity of mobile phase was increased, the degree of separation between monosaccharide derivatives will also be improved. We compared the separation effects of PBS and acetonitrile-ammonium formate (including formic acid), and the results showed that when PBS was mixed with organic phase, phosphate would precipitate, resulting in unstable pressure of liquid chromatography system. When ammonium formate-formic acid buffer was used, the pressure of liquid chromatography system was stable and the peak type was good, which was suitable for the determination of monosaccharide derivatives.

### 3.3 Optimization of extraction conditions

**Figure 2:**
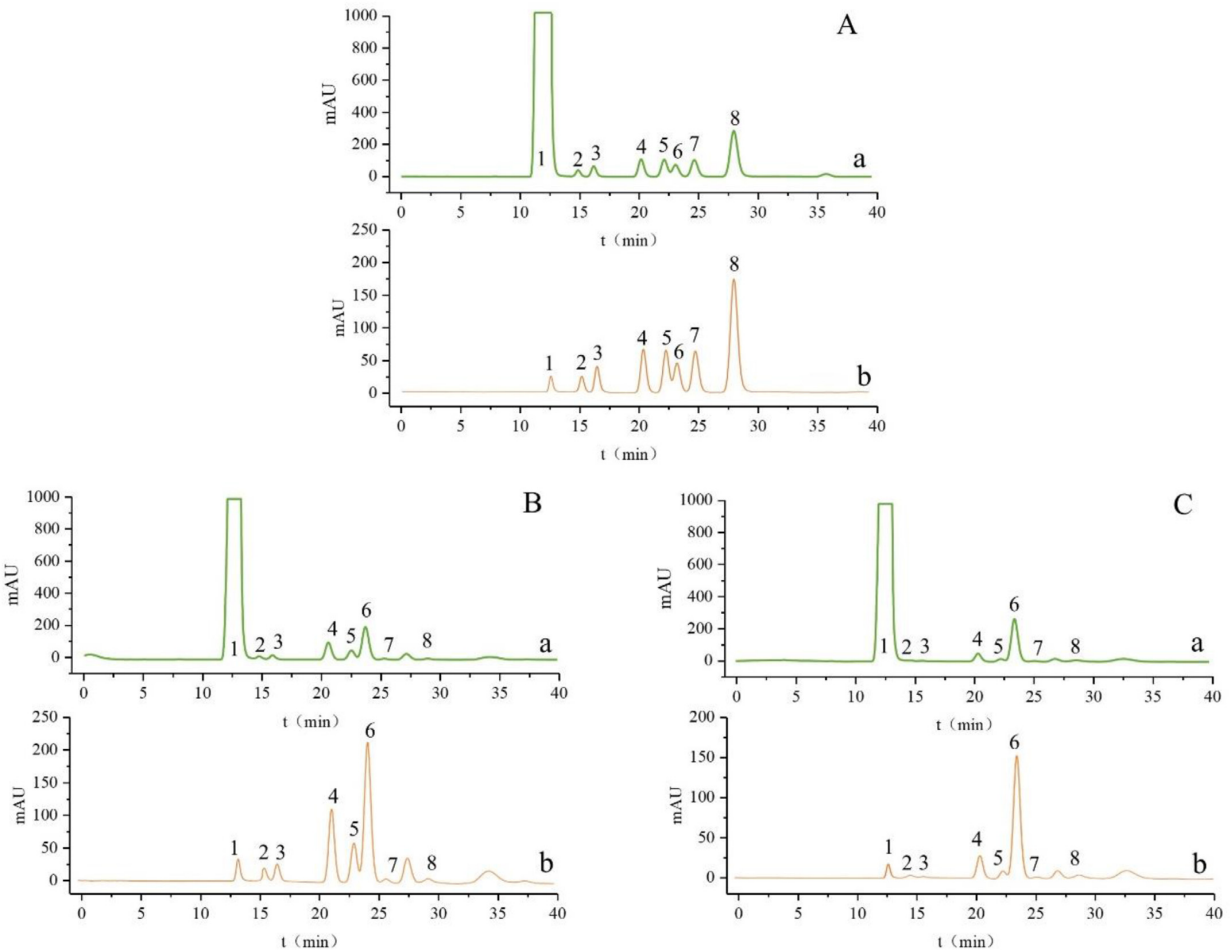
Chromatogram before and after extraction of derivative products. (A) mixed reference substance (B) *Polygonatum sibiricum* sample (C) *Polygonatum odoratum* sample before extraction (a) and after extraction (b). 1. PABA, 2. fructose, 3. rhamnose, 4. galactose, 5. glucose, 6. mannose, 7. xylose, 8. galacturonic acid.

In this experiment, the physical properties of PABA were reviewed, and it was found that the solubility of PABA in ethyl acetate was higher than that in water. Considering that the peak of derivative reagent would affect the assignment of monosaccharide derivative peak, and the peak of monosaccharide derivative may overlap with the peak of derivative reagent. The test solution prepared according to the analysis method of reference [25] would have a large number of crystals precipitated after being placed at room temperature for 4 h, and the chromatogram contained high derivative reagent peaks, which affected the determination results. Ethyl acetate was insoluble in water. When the two liquids were mixed, ethyl acetate was in the upper layer and water was in the lower layer. After extraction with ethyl acetate, the content of PABA in the test solution decreased obviously. As shown in Figure 2, after extraction with ethyl acetate, the chromatogram of derivative reagent was obviously reduced, and the interference of derivative reagent peak was removed. The solution remained stable after 24 h at room temperature, indicating that ethyl acetate had good extraction efficiency on derivative reagents.

### 3.4 Methodology

#### 3.4.1 Linear equation, correlation coefficient, LOQ and LOD

The standard solution was diluted to a certain gradient concentration before derivatization determination was performed separately. The linear equation and correlation coefficient of each monosaccharide are shown in Table 2. Each monosaccharide had good linear relationship in its linear range, and the correlation coefficient was greater than 0.997. The limit of quantification was between 0.29–0.64 μg/mL, and the limit of detection was between 0.12–0.36 μg/mL. The established analytical method had low LOQ and LOD, indicating that this method was sensitive and suitable for analysis and determination of monosaccharides.

**Table 2:** Methodological investigation results of seven monosaccharides.

Monosaccharide	Linear equation	correlation coefficient	Linearity range(μg/mL)	LOQ(μg/mL)	LOD(μg/mL)	RSD%			Recovery %	
						Precision	Repeatability	Stability	Average	RSD
Fru	y = 455.24x + 332.6	0.9981	1–100	0.64	0.28	2.3	3.2	1.6	98.94	2.5
Rha	y = 840.86x + 561.01	0.9979	1–100	0.55	0.36	1.5	1.3	2.5	101.25	3.1
Gal	y = 20250x – 2786.2	0.9997	0.5–50	0.41	0.28	2.7	4.3	2.4	102.52	3.4
Glc	y = 20041x – 1524.3	0.9999	0.5–50	0.33	0.19	1.8	3.5	2.6	97.63	2.6
Man	y = 15090x – 2052.7	0.9998	0.5–50	0.48	0.30	3.2	2.9	3.5	99.56	3.7
Xyl	y = 21223x – 1561.9	0.9999	0.5–50	0.29	0.12	1.6	3.8	3.9	98.29	2.5
GalUA	y = 6281.9x – 1055.2	0.9985	0.5–50	0.47	0.34	2.9	3.2	3.3	98.34	2.7

#### 3.4.2 Precision, repeatability, stability and recovery

The derivatives prepared by this method were tested for precision, repeatability, stability and sample recovery. The experimental results were shown in Table 2. The RSD of precision was between 1.5%–3.2% and the RSD of repeatability was between 1.3%–4.3%, indicating that the method had high precision and good repeatability. The RSD of stability was between 1.6%–3.9%, indicating that the sample was stable at room temperature for 12 h. The recovery was between 97.63%–102.52%, indicating that the determination results were accurate. The established method was suitable for the determination of monosaccharides in *Polygonatum sibiricum* and *Polygonatum odoratum*.

### 3.5 Determination result of samples

Dry samples of *Polygonatum odoratum* and *Polygonatum sibiricum* were measured according to the above method, and the measurement results were shown in Table 3. Both *Polygonatum sibiricum* and *Polygonatum odoratum* contained seven monosaccharides, and the monosaccharide content in *Polygonatum sibiricum* was higher than that in *Polygonatum odoratum*. The monosaccharide content in the roots of *Polygonatum sibiricum* was higher than that in the *Polygonatum sibiricum* fibrous roots, the monosaccharide content in the roots of *Polygonatum odoratum* was higher than that in the *Polygonatum odoratum* fibrous roots. The monosaccharide content in main roots of *Polygonatum sibiricum* and *Polygonatum odoratum* was higher than that in fibrous roots, which was basically consistent with the research results in references [28, 29]. Mannose was the main monosaccharide component in *Polygonatum sibiricum* and *Polygonatum odoratum*. The content of fructose in *Polygonatum sibiricum* and *Polygonatum odoratum* was the lowest, while the contents of fructose and galacturonic acid in the *Polygonatum odoratum* fibrous roots and *Polygonatum odoratum* fibrous roots were lower, the results indicated that the monosaccharide contents in roots and fibrous roots of *Polygonatum sibiricum* and *Polygonatum odoratum* were quite different. The experimental results showed that the fibrous roots of *Polygonatum sibiricum* and *Polygonatum odoratum* also contained a certain amount of polysaccharides, proving that their fibrous roots also had certain medicinal value.

**Table 3:** Determination results of monosaccharide content in *Polygonatum sibiricum* and *Polygonatum odoratum*.

Sample	Fru(mg/g)	Rha(mg/g)	Gal(mg/g)	Glc(mg/g)	Man(mg/g)	Xyl(mg/g)	GalUA(mg/g)	Total(mg/g)
*Polygonatum sibiricum* l	0.3685	1.3229	1.4588	0.2381	4.5244	0.7852	1.2061	9.9040
*Polygonatum sibiricum* 2	0.4358	1.0530	1.5227	0.2679	4.7679	0.7357	1.3374	10.120
*Polygonatum sibiricum* 3	0.2441	0.7991	0.7519	0.2715	5.2839	0.1074	0.7609	8.2188
*Polygonatum sibiricum* 4	0.5081	1.4991	1.4006	0.7294	3.7996	0.1139	1.4918	9.5425
*Polygonatum sibiricum* 5	0.4052	1.1386	1.3254	0.4329	4.1398	0.4539	1.0394	8.9352
*Polygonatum sibiricum* 6	0.3461	1.0580	1.2863	0.2653	4.5486	0.4376	1.0527	8.9946
*Polygonatum odoratum* 1	0.1033	0.1834	1.1140	0.2205	1.9528	0.4008	0.152	4.1268
*Polygonatum odoratum* 2	0.0976	0.1228	1.1268	0.2048	1.9930	0.3536	0.1493	4.0479
*Polygonatum odoratum* 3	0.1235	0.1591	0.5046	0.1929	2.0566	0.0771	0.0353	3.1491
*Polygonatum odoratum* 4	0.1059	0.1458	0.7969	0.1252	0.8091	0.0763	0.2206	2.2798
*Polygonatum odoratum* 5	0.1026	0.1134	0.6817	0.1573	1.3249	0.3255	0.1855	2.8909
*Polygonatum odoratum* 6	0.1203	0.1028	0.9395	0.1654	1.4216	0.1853	0.1436	3.0785
*Polygonatum sibiricum* fibrous roots 1	0.1472	0.8333	0.5026	0.4027	0.8245	0.1253	0.0263	2.8619
*Polygonatum sibiricum* fibrous roots 2	0.1835	0.6923	0.5107	0.5385	0.7526	0.1337	0.0286	2.8399
*Polygonatum sibiricum* fibrous roots 3	0.2342	0.7531	0.4853	0.4953	0.7155	0.1654	0.0309	2.8797
*Polygonatum sibiricum* fibrous roots 4	0.1593	0.8146	0.4378	0.3759	0.8064	0.1429	0.0428	2.7797
*Polygonatum sibiricum* fibrous roots 5	0.1624	0.8007	0.6537	0.4855	0.8526	0.1833	0.0192	3.1574
*Polygonatum sibiricum* fibrous roots 6	0.1450	0.9628	0.7284	0.6038	0.7219	0.1437	0.0319	3.3375
*Polygonatum odoratum* fibrous roots 1	0.0193	0.0991	0.216	0.1202	0.1493	0.0773	0.0184	0.6996
*Polygonatum odoratum* fibrous roots 2	0.0185	0.0789	0.2083	0.1033	0.2016	0.0709	0.0153	0.6968
*Polygonatum odoratum* fibrous roots 3	0.0163	0.0835	0.3186	0.0998	0.202	0.0684	0.0192	0.8078
*Polygonatum odoratum* fibrous roots 4	0.0174	0.1200	0.2647	0.1135	0.1638	0.0849	0.0127	0.7770
*Polygonatum odoratum* fibrous roots 5	0.0208	0.1054	0.2354	0.1534	0.1547	0.0857	0.0146	0.7700
*Polygonatum odoratum* fibrous roots 6	0.0210	0.1034	0.1476	0.1429	0.1213	0.0789	0.0154	0.6305

### 3.6 Chemometric analysis of monosaccharide composition in *Polygonatum sibiricum* and *Polygonatum odoratum*

**Figure 3:**
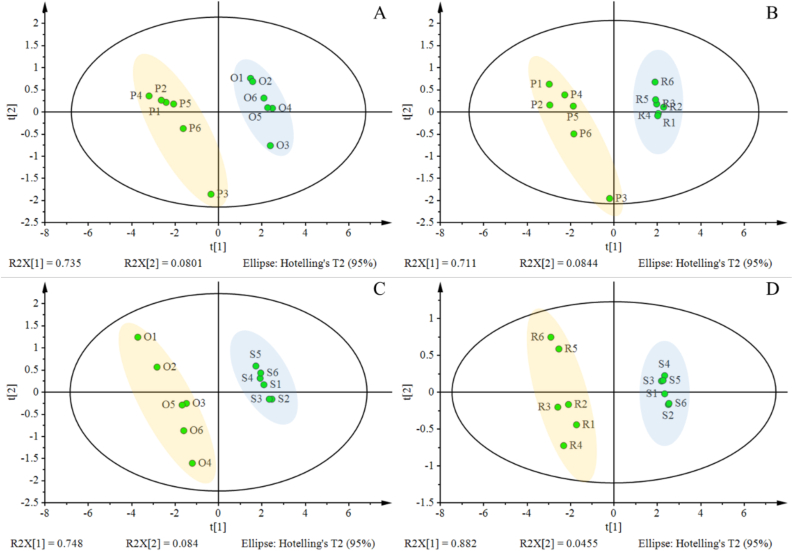
PLS-DA score diagram of monosaccharide composition in *Polygonatum sibiricum* root sample (P) *Polygonatum odoratum* root sample (O) *Polygonatum sibiricum* fibrous roots (R) and *Polygonatum odoratum* fibrous roots (S). Four different samples were used: (A) *Polygonatum sibiricum* and *Polygonatum odoratum*, (B) *Polygonatum sibiricum* and *Polygonatum sibiricum* fibrous roots, (C) *Polygonatum odoratum* and *Polygonatum odoratum* fibrous roots, (D) *Polygonatum sibiricum* fibrous roots and *Polygonatum odoratum* fibrous roots.

The samples of *Polygonatum sibiricum*, *Polygonatum odoratum* and their corresponding fibrous roots were processed according to the above analysis method, and the monosaccharide content in *Polygonatum sibiricum* and *Polygonatum odoratum* was calculated respectively, and the difference of monosaccharide composition in different samples was compared. The results of sample determination were analyzed by principal component analysis using Simca13.0 software and calculated by PLS-DA model. As shown in Figure 3, there was obvious difference in monosaccharide content between *Polygonatum sibiricum* and *Polygonatum sibiricum* fibrous roots, and there was obvious difference in monosaccharide content between *Polygonatum odoratum* and *Polygonatum odoratum* fibrous roots. By comparing the VIP values of different types of samples, the monosaccharide component with the highest VIP score was used as the distinction between the two types of samples. Mannose was the most important distinguishing component in the *Polygonatum sibiricum* and *Polygonatum odoratum*, *Polygonatum sibiricum* and *Polygonatum sibiricum* fibrous roots, *Polygonatum sibiricum* fibrous roots and *Polygonatum odoratum* fibrous roots. Fructose was the most important distinguishing component between *Polygonatum odoratum* and *Polygonatum odoratum* fibrous roots. Using PLS-DA analysis, the monosaccharide composition could be better distinguished between the *Polygonatum sibiricum* and *Polygonatum odoratum*, *Polygonatum sibiricum* and *Polygonatum sibiricum* fibrous roots, *Polygonatum sibiricum* fibrous roots and *Polygonatum odoratum* fibrous roots, *Polygonatum odoratum* and *Polygonatum odoratum* fibrous roots.

In this study, an HPLC-FLD method was established to determine monosaccharide contents in *Polygonatum sibiricum* and *Polygonatum odoratum*. The fluorescence derivatization method of monosaccharide was first applied to the detection of polysaccharides in Chinese medicinal materials. The method had high precision, good repeatability and accurate determination results. Compared with ultraviolet detection of monosaccharide, fluorescence detection method had higher detection sensitivity and lower detection cost than mass spectrometry. The limit of quantification was between 0.29–0.64 μg/mL, and the limit of detection was between 0.12–0.36 μg/mL. HPLC-FLD had high sensitivity in detecting PABA derivatives of monosaccharides. The analysis of seven monosaccharides in *Polygonatum sibiricum* and *Polygonatum odoratum* could be completed within 30 min. The analysis time was shorter, which was suitable for the determination of monosaccharides in *Polygonatum sibiricum* and *Polygonatum odoratum*. Considering various factors, the method in this study was superior to other methods in similar studies at present, as shown in Table S1 in the Supplementary Material.

A pretreatment method of *Polygonatum sibiricum* polysaccharide was established in this study. Fluorescent markers of monosaccharides were prepared by PABA derivatization. Because the solubility of PABA in water was different from that in ethyl acetate, ethyl acetate could be used to extract PABA, thus reducing redundant derivatization reagents in samples and improving detection sensitivity. Compared with the analysis method in reference [25], the chromatographic peaks of derivative reagents in unextracted samples were high in intensity, which easily covered the chromatographic peaks of monosaccharide derivatives. Comparing the peak conditions of samples before and after extraction, the results showed that ethyl acetate was effective in extracting PABA from samples. After extraction, it could significantly reduce the amount of derivative reagents in samples and retain the target components, which was suitable for the determination of monosaccharide content in *Polygonatum sibiricum* and *Polygonatum odoratum*.

In this study, orthogonal experiments were used to optimize the PABA derivatization conditions of monosaccharides. It was determined that the best derivatization effect was obtained when the derivatization reagent was 200 μL, the derivatization temperature was 70 °C and the derivatization time was 40 min. After extracting monosaccharide derivatives with ethyl acetate, the peak of derivative reagent of the test sample could be obviously reduced, and the interference of derivative reagent on the determination results could be reduced. The determination results of *Polygonatum sibiricum* and *Polygonatum odoratum* showed that mannose was the highest monosaccharide content in *Polygonatum sibiricum* and *Polygonatum odoratum*. Compared with the content of monosaccharide in roots and fibrous roots, the content of monosaccharide in main roots was higher than that in fibrous roots, which might be related to the transport of polysaccharides in plants and the role of microorganisms around roots [30, 31]. The amount of polysaccharide transported from roots to fibrous roots in plants was small, while the growth of microorganisms around roots consumed more, leading to decreased polysaccharide content in fibrous roots, especially in the tip of roots. In this study, the monosaccharide derivatized from PABA was applied to the analysis of the composion of Chinese medicinal materials. The derivatization conditions of monosaccharide were optimized, and the extraction method that could be used to reduce the chromatographic peaks of derivatization reagents was provided. This study will provide new knowledge for the fluorescence analysis of monosaccharide in Chinese medicinal materials. The difference of polysaccharide content between *Polygonatum sibiricum* and *Polygonatum odoratum* and their corresponding fibrous roots could provide reference for the chemical composition of *Polygonatum sibiricum* and *Polygonatum odoratum*, for future development and utilization of the medicinal components and medicinal parts of *Polygonatum sibiricum* and *Polygonatum odoratum*, and for promoting healthy, stable and sustainable development of the natural medicine industry of *Polygonatum sibiricum* and *Polygonatum odoratum*.

## Declarations

### Author contribution statement

Sheng Xu, Jianli Bi: Performed the experiments.

Wenfang Jin: Contributed reagents, materials, analysis tools or data.

Baolei Fan: Conceived and designed the experiments; Wrote the paper.

Chunqi Qian: Analyzed and interpreted the data; Wrote the paper.

### Funding statement

This work was supported by Key Projects of Hubei 10.13039/100009210Food and Drug Administration (20180103), Traditional Chinese Medicine Research Projects of Hubei Health Commission (ZY2019M029) and General Program of Hubei Health Commission (2019-20YZ07).

### Data availability statement

Data included in article/supplementary material/referenced in article.

### Declaration of interests statement

The authors declare no conflict of interest.

### Additional information

No additional information is available for this paper.

## Appendix A Supplementary data

SupplementalMaterial SupplementalMaterial
