# Supplementary material for: Determination of polysaccharides composition in Polygonatum sibiricum and Polygonatum odoratum by HPLC-FLD with pre-column derivatization
Source: Heliyon. 2022 Apr 30;8(5):e09363. doi: 10.1016/j.heliyon.2022.e09363 (PMC9109187; doi:10.1016/j.heliyon.2022.e09363)
Supplement: SupplementalMaterial [file mmc1.docx]

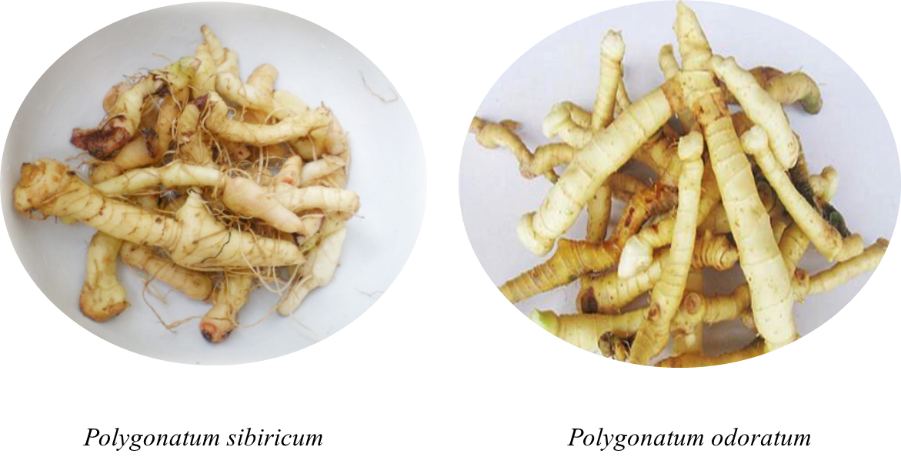


**Fig. S1 Medicinal parts of samples of *Polygonatum sibiricum*** **and *Polygonatum odoratum* at same age**


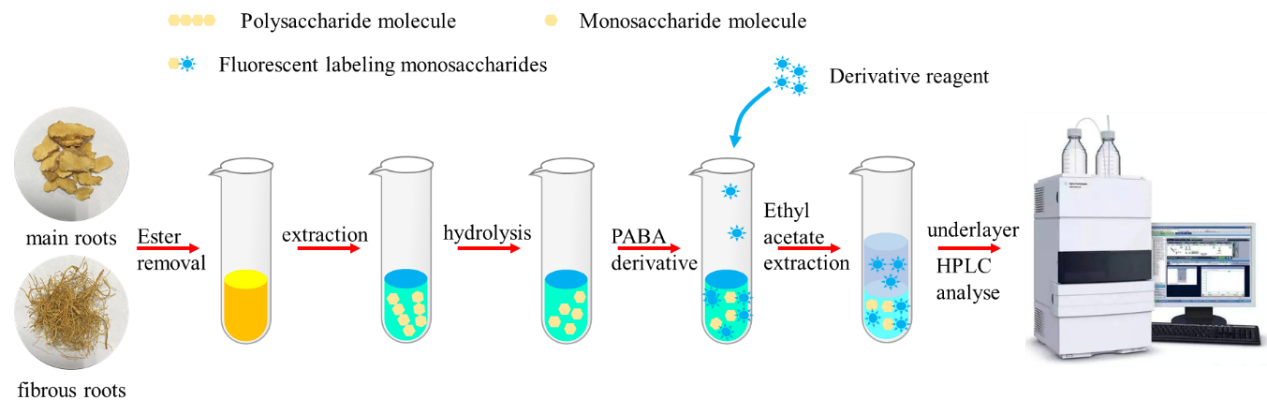


**Fig. S2 Pretreatment method of main root and fibrous roots samples**


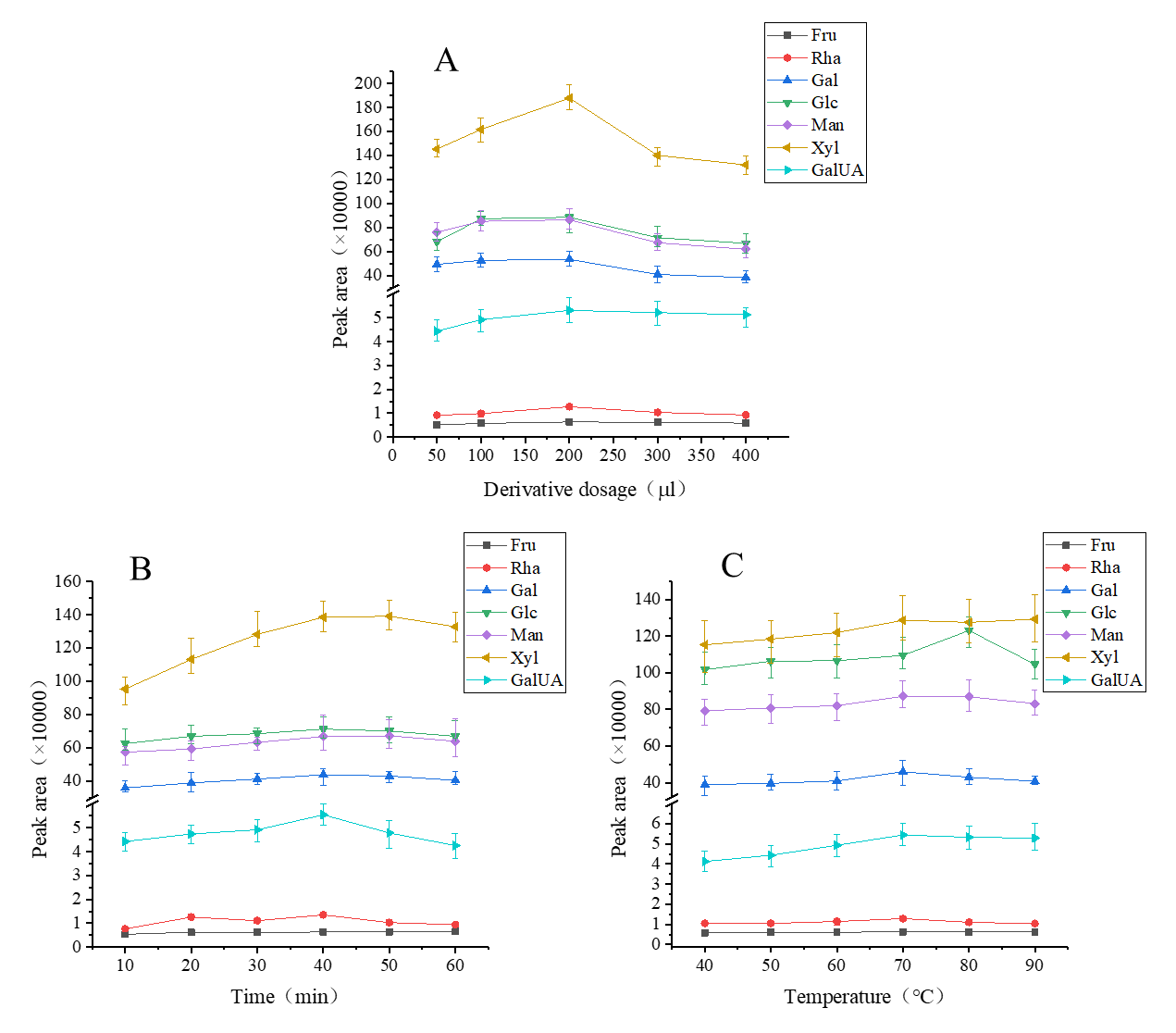


**Fig. S3. Chromatographic peak intensity of derivative products under different derivatization conditions**

**A. different derivatization reagent dosage B. different derivatization time C. different derivatization temperature**

**Table S1 Comparison of different detection methods of polysaccharides**

| Monosaccharide | Method | Time | LOQ  μg/ml | LOD  μg/ml | Reference |
| --- | --- | --- | --- | --- | --- |
| Rha, GlcUA, Gla, Ara, Fuc, Man, Glc, Rib, Xyl | HPLC-UV | 80 min | 49.88-67.125 | 1.11-2.58 | [15] |
| Rha, Fuc, Ara, Xyl, Man, Glu, Gal, GlcUA, GalUA | GC-FID | 45 min | 25-30 | 0.27-1.65 | [17] |
| Gal, Glu, Man, Fru, Xyl, Rib | CEZ | 60 min | 5-50 | 3-25 | [18] |
| Man, Rib, Glu, Gal, Xyl, Fuc | HPLC-MS | 30 min | 0.18-0.60 | 0.06-0.20 | [20] |
| Fru, Rha, Gal, Glc, Man, Xyl, GalUA | HPLC-FLD | 30 min | 0.29-0.64 | 0.12-0.36 | This study |
